# Rectosigmoid sparing en bloc pelvic resection for fixed ovarian tumors: Surgical technique and perioperative and oncologic outcomes

**DOI:** 10.3389/fonc.2022.980050

**Published:** 2022-08-22

**Authors:** Ying Shan, Ying Jin, Yan Li, Yu Gu, Wei Wang, Lingya Pan

**Affiliations:** Department of Obstetrics and Gynecology, National Clinical Research Center for Obstetric and Gynecologic Diseases, Peking Union Medical College Hospital, Chinese Academy of Medical Sciences and Peking Union Medical College, Beijing, China

**Keywords:** rectosigmoid sparing, en bloc pelvic resection, ovarian cancer, surgical technique, tumorectomy

## Abstract

**Purpose:**

Patients with advanced ovarian cancer often undergo en bloc rectosigmoid resection with total hysterectomy to completely debulk the pelvis. We describe a unique rectosigmoid sparing en bloc pelvic resection technique for fixed ovarian tumors infiltrating the colon wall.

**Methods:**

From July 2020 to June 2021, 20 patients with advanced epithelial ovarian cancer (EOC) underwent rectosigmoid sparing en bloc pelvic resection successfully at our institution. We summarized our surgical technique and the peri-operative and oncological outcomes.

**Results:**

Twenty cases with bowel infiltration achieved en bloc pelvic resection with rectosigmoid tumorectomy in a centripetal fashion. Only two patients required mucosal repair. None of the patients experienced any complications associated with en bloc resection. No pelvic recurrence occurred within the median follow-up time of 12 months.

**Conclusion:**

Rectosigmoid sparing en bloc pelvic resection may be feasible for select patients with fixed ovarian tumors infiltrating the colon wall.

## Introduction

Ovarian cancer is the most lethal of all gynecologic malignancies. Surgery with complete residual tumor removal (R0 resection) is the recommended treatment and has the greatest prognostic impact (1, 2). To obtain complete cytoreduction, patients with advanced ovarian cancer often undergo en bloc rectosigmoid resection with total hysterectomy to completely debulk the pelvis. Hudson published the first report describing this technique, termed “radical oophorectomy”, which was specifically designed for the intact removal of a fixed ovarian tumor en bloc along with the attached peritoneum and surrounding structures (3). Since then, it has been adopted by many medical institutions around the world (4–9). The rectosigmoid colon is frequently involved in these cases, and rectosigmoid colon resection is performed in 25%–58% of all patients (5, 10–13). Anastomotic leakage is the most feared complication. Common complications are persistent urinary, defecatory, and sexual dysfunction due to autonomic nervous system damage arising from surgery (13). However, considering that epithelial ovarian cancer (EOC) tumors usually involve part of the wall of the colon and are limited to the serosa in 45% of cases (14), ulceration into the rectum is very rare (15). Moreover, since the goal of debulking surgery is the complete removal of macroscopic neoplasms, not radical resection, it is feasible to perform an en bloc pelvic resection with tumorectomy for tumors that are fixed in the pelvis and infiltrate the rectosigmoid colon, avoiding colectomy. The purpose of this paper is to describe a rectosigmoid sparing en bloc pelvic resection technique for fixed ovarian tumors infiltrating the colon wall. We summarize our surgical technique and the peri-operative and oncological outcomes.

## Materials and methods

From July 2020 to June 2021, among the patients who underwent primary or interval debulking surgery, 20 patients with advanced EOC received rectosigmoid sparing en bloc pelvic resection successfully at the Department of Gynecologic Oncology Peking Union Medical College Hospital. Relative contraindications to the procedure include a Gynecologic Oncology Group performance status score of ≥3 and/or a tumor distribution that precludes an attempt to achieve complete resection, namely, extensive tumor infiltration of the small bowel mesenteric root, unresectable involvement of the porta hepatis, large-volume (≥1 cm) unresectable extra-abdominal metastasis (e.g., pulmonary), or multiple unresectable parenchymal liver metastases.

**Figure 1:**
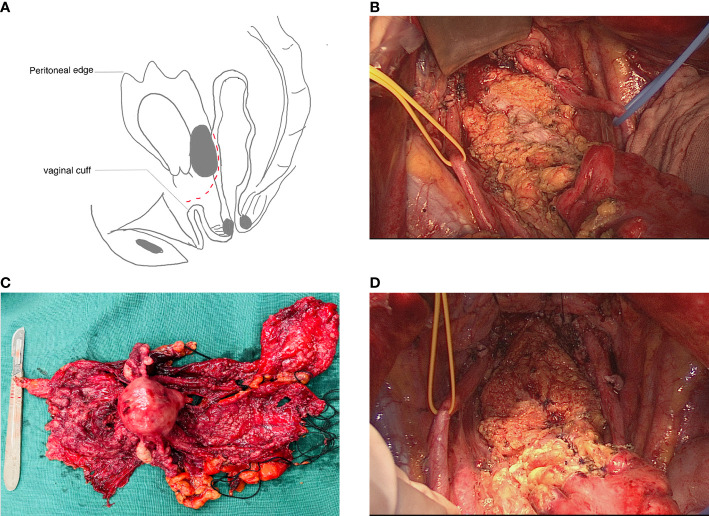
Rectosigmoid sparing en bloc pelvic resection for fixed ovarian tumors. **(A)** Tumor attaching the intact specimen was left on the colon as the bottom of the false capsule. Cutting plane is shown as a dashed line. **(B)** Seromuscular defections (black arrow) after complete resection of implants on the rectosigmoid colon. **(C)** The whole specimen was removed intact with a false capsule. **(D)** Seromuscular layer was repaired with interrupted sutures.

The surgical procedures are shown in **Table 1**. Steps such as retroperitoneal exposure, infundibulopelvic ligament ligature, ureterolysis, uterine artery ligature, retrograde hysterectomy, and retrograde rectovaginal septum dissection have been described in many reports on Hudson procedures. These procedures were accomplished using a retroperitoneal approach. The para-rectal and presacral spaces were not intentionally exposed. After rectovaginal septum dissection and tumor-involved mesosigmoid and mesorectal peritoneum shaving, the entire segment of the affected peritoneum and uterus was dissected and removed as a part of the false capsule. Since a tumor that is fixed to the Douglas pouch can infiltrate the rectosigmoid colon, in these cases, the attached tumor held in place by the rectosigmoid colon was left on the colon to serve as the bottom of the false capsule, as shown in **Figure 1A**. At this time, evaluations were made by experienced surgeons before tumorectomy. If tumorectomy led to laceration of <30%–40% of the colon wall or if the defect in the seromuscular layer was not too extensive, patients received rectosigmoid sparing surgery. Otherwise, en bloc rectosigmoid resection was performed, because if the area of the colonic defect was too extensive, there would be a high risk of colon fistula or stricture after repairment. Tumorectomy was performed in a centripetal fashion with a monopolar device. After complete resection of the tumor held by the rectosigmoid colon (**Figure 1B**), the whole specimen was removed intact with the false capsule (**Figure 1C**). The seromuscular layer was repaired with interrupted sutures; sometimes sutures perpendicular to the long axis of the bowel were not required (**Figure 1D**). The two-layer repair was performed if mucosal defects were observed.

**Table 1:** Surgical steps of rectosigmoid sparing en bloc pelvic resection.

1. Pelvic parietal peritoneum dissection, accession to the retroperitoneal space 2. Ligation of the infundibulo-pelvic ligament 3. Isolation of the ureter laterally 4. Retrograde mobilization of the bladder peritoneum with access to the vesico-vaginal space 5. Ligation of uterine vessels at the level of ureter and parametrial resection 6. Colpotomy 7. Recto-vaginal septum dissection 8. Tumor involved mesorectal and mesosigmoid peritoneum shaving 9. Rectosigmoid tumorectomy in a centripetal fashion and bowl defects repairment

## Results

The median age of the patients was 62 years (range 28–75 years). High-grade serous ovarian cancer (HGSOC) was the predominant histological type and was detected in 16 of 20 (80%) patients. The most common International Federation of Gynecology and Obstetrics (FIGO) stage was IIIC, which was confirmed in 90% of patients. Detailed data on the preoperative characteristics are shown in **Table 2**. Intraoperative and postoperative outcomes are shown in **Table 3**. Optimal debulking was achieved in all patients; 19 of 20 achieved R0 (no residual tumor), and only one had a residual tumor less than 2 mm on the mesentery of the small intestine. The median duration of the procedure was 230 min (range 175–340 min). The time interval from surgery to the start of chemotherapy was 9 days (range 7–13 days). The surgical complexity score (introduced by Aletti) (16) of 20 patients was 6–9; instead of rectosigmoidectomy and anastomosis, tumorectomy accounted for 3 points in this system since they shared many of the same steps. The median estimated blood loss (EBL) was 500 cc (range 300–1,200 cc). No tumors were observed to infiltrate the mucosa, and interrupted repair of the seromuscular layer was performed in 18 patients. The mucosal repair was performed, and total parenteral nutrition was provided to two patients (10%) because of a mucosal defect caused by tumorectomy. The size of the solid tumor fixed in the Douglas pouch was measured by CT. The median length of the long axis was 51 mm (range 36–68 mm), and the median length of the bowel with a serosal defect was 10 cm (range 3–20 cm).

**Table 2:** The clinical characteristics of the included patients.

Variable	Value/no. of patients (N = 20)
**Median age (years)**	62 (range 28–75)
**Type of surgery**	
PDS	15
IDS	5
**Tumor histology**	
HGSOC	16
LGSOC	2
Musinous	2
**FIGO stage**	
IIIc	18
IVA	2

PDS, primary debulking surgery; IDS, Interval debulking surgery; HGSOC, high-grade serous ovarian carcinoma; LGSOC, low-grade serous ovarian carcinoma; FIGO, International Federation of Gynecology and Obstetrics.

**Table 3:** Intra-operative and post-operative outcomes.

Patients no.	Surgery duration (min)	Tumor size (mm)	EBL (cc)	SCS	Length of seromuscular defection (cm)	Mucosal layer repairment	Residual disease	TPN (days)	Overall morbidity	Time between surgery and Cht (days)	Follow-up time (months)	Sites of recurrence
1	175	57 * 28	400	6	3	No	R0	0		8	18	–
2	270	43 * 25	500	7	8	No	R0	0		9	17	–
3	240	43 * 30	600	7	5	No	R0	0		7	16	–
4	340	63 * 32	1200	8	6	Yes	R0	4		13	15	Inguinal lymph nodes
5	280	56 * 40	900	8	10	Yes	R1	4		11	15	–
6	195	42 * 29	300	7	11	No	R0	0		9	14	–
7	210	39 * 28	350	8	9	No	R0	0		8	13	–
8	195	49 * 24	500	6	15	No	R0	0		8	13	–
9	240	43 * 31	400	7	12	No	R0	0		9	13	–
10	330	39 * 24	1100	9	6	No	R0	0	Pleural effusion	7	13	–
11	285	36 * 32	600	7	8	No	R0	0	DVT	9	12	–
12	320	63 * 53	800	7	12	No	R0	0		10	12	–
13	190	44 * 30	400	7	13	No	R0	0		9	11	–
14	180	53 * 39	500	8	8	No	R0	0		10	10	–
15	215	67 * 38	300	8	17	No	R0	0		8	10	–
16	230	48 * 45	450	8	3	No	R0	0		9	10	–
17	240	57 * 47	850	9	7	No	R0	0	Pleural effusion	8	9	–
18	220	68 * 57	550	7	9	No	R0	0		7	8	–
19	230	59 * 53	450	7	11	No	R0	0		8	8	–
20	225	67 * 39	600	6	20	No	R0	0		8	7	–

Tumor size, size of tumor in Douglas pouch measured in CT; SCS, surgical complexity score; EBL, estimated blood loss; TPN, parenteral nutrition; PDS, primary debulking surgery; Cht, chemotherapy.

*Means by multiply.

No patient experienced complications associated with en bloc resection, and no stricture or subsequent obstruction occurred after bowel repair. Two patients had pleural effusion because of diaphragmatic peritoneal stripping, and one had deep vein thrombosis (DVT). There was no readmission within 30 days and no surgery-related deaths.

After surgery, all patients were administered six cycles of standard adjuvant chemotherapy (carboplatin area under curve 5 and paclitaxel 175 mg/m^2^). The time interval from surgery to the start of chemotherapy was 9 days (range 7–13 days). No pelvic recurrence occurred during the median follow-up time of 12 months.

## Discussion

En bloc pelvic resection of ovarian cancer with fixed tumors in the pelvis was first reported by Hudson 60 years ago (3). In principle, this technique consists of the removal of the entire Douglas pouch, serving as a false capsule of the tumor. The cardinal feature of this procedure is the approach to the retroperitoneal space, in which extensive intraperitoneal tumors are not involved, so dissection can be performed in a centripetal fashion, ensuring maximum safety to the surrounding vital structures, particularly if the pelvic organs can no longer be clearly identified.

When bowel infiltration is suspected, en bloc resection of the rectosigmoid colon is the most frequently performed variation of the Hudson procedure (4, 6, 8, 9, 17–21). Indications for rectosigmoid resection (RR) vary across centers. For instance, RR has been indicated for extensive involvement of the cul-de-sac and rectosigmoid colon in some reports (7), while it has been indicated for bowel wall infiltration even in cases with only superficial involvement in other reports (13, 14). Considering the complications that can occur after RR, rectosigmoid mesorectal-sparing resection can maximize the blood supply to colorectal anastomosis and minimize the risk of both anastomotic leakage and pelvic autonomic nervous system dysfunction (22). However, there is still the risk of anastomotic leakage.

If the tumor penetrates the muscularis of the colon but has a limited (≤2 cm) longitudinal extent, a full-thickness “wedge-shaped” segment of the anterior rectal wall can be sharply excised. Plotti (23) reported a similar method for partial rectosigmoid resection, which was performed when the complete removal of the disease led to laceration of <30%–40% of the intestinal wall circumference. The oncologic outcomes of 5-year overall survival and optimal debulking rates were not significantly different from those obtained with total rectosigmoid resection. This more conservative approach to the rectum seems to be a feasible approach for over 40% of patients with advanced ovarian cancer and rectosigmoid colon involvement.

However, it should be noted that unlike colon cancer, it is very rare for ovarian tumors to ulcerate into the rectum. Histopathological findings from the main studies on primary debulking with RR in patients with advanced ovarian cancer (AOC) have revealed superficial infiltration in a very large percentage of cases, with the infiltration being limited to the serosa or subserosa in 28%–71% of cases. Based on this view, some studies have asserted that conservative ablation may be safe and effective, like RR (14). However, the criterion of no gross residual tumor is preferred for optimal cytoreduction instead of R0 resection in these patients.

Unlike radical surgery, debulking surgery aims to achieve complete resection of all visible diseases (24–28), and considering that there is a boundary between the solid tumor and bowel wall, it is feasible to achieve complete resection in a rectosigmoid sparing fashion. Kim et al. (29) published the first report on the impact of tumorectomy without bowel resection for affected rectosigmoid lesions on EOC survival outcomes and operation-related morbidity. Their results revealed that the survival outcomes of patients treated with tumorectomy were not inferior to those of patients treated with RR if optimal debulking could be guaranteed. However, notably, the cohort excluded patients who had rectosigmoid lesions infiltrating up to the muscle, and the tumorectomy procedure used was a kind of serosectomy technique performed in patients with superficial bowel infiltration.

We developed a novel technique that achieved complete resection with tumorectomy in an en bloc manner for tumors fixed in the pelvis, even some with seromuscular infiltration, in which case rectosigmoid resection was required using the traditional Hudson procedure.

Since it may be difficult to distinguish the severity of bowel infiltration, the most important decision regarding whether rectosigmoid sparing or not was not made at the beginning of the surgery; this is the major difference compared with several modified en bloc resection planned anastomosis procedures, in which rectosigmoid bowel division is performed at the start of the surgery.

Sometimes tumors fixed in the pelvis seemed to infiltrate the bowel wall in the Douglas pouch, but only peritoneum and part of mesocolon involvement were found after peritoneum shaving. Hertel et al. (13) reported that bowel infiltration was not found on histopathologic examination in 27% of patients who underwent RR. For these patients who had exclusive involvement of the cul-de-sac but no bowel infiltration, retrograde hysterectomy and excision of the involved peritoneum in an en bloc manner should be performed without bowel resection. Moreover, extensive disease in the peritoneum and tumors fixed in the pelvis may cause the colon to be distorted or folded such that the severity of bowel infiltration cannot be evaluated objectively until retrograde rectovaginal septum dissection and mesosigmoid and mesorectal peritoneum shaving are complete. After this step, only the tumor as the bottom of the false capsule remained on the rectosigmoid colon, so the severity of bowel invasion could be visualized clearly. At this time, evaluation and decisions regarding rectosigmoid sparing can be made by experienced surgeons.

Tumorectomy is the most unique part of our procedure, and several points should be considered to accomplish it successfully. First, to achieve complete resection and avoid cutting through the tumor tissue, the cutting plane should be on the healthy part beneath the border of the tumor and the normal colon, as shown in **Figure 1A**. Sacrificing complete resection for an intact bowel wall is not the goal of the procedure. Second, since there is a high risk of mucosal defect if cutting is performed too rapidly with a high-level electrical device; thus, setting the monopolar device to a moderate setting and keeping the device moving while identifying the cutting plane will minimize the electrical injury and carbonization of normal tissue. Third, to minimize mucosal defects, tumorectomy should be performed in a centripetal fashion (the point of the tumor that infiltrates deepest into the bowel wall was regarded as the “center”). In our case, since the tumor infiltrated irregularly into the seromuscular layer of the bowel, the cutting route had irregular lines, which should be continually adjusted to identify the relatively loose space in the muscular layer beneath the tumor, leaving the part of the bowel with the deepest tumor infiltration to be separated at the end of the resection. Once mucosal perforation occurs, the best cutting plane will be lost, and the bowel still attached to the tumor must be removed in a full-thickness fashion, causing more mucosal defects, which may cause the procedure to be converted to RR. Proper tension perpendicular to the cutting plane will make it easier to find the cutting plane. However, retracting the tumor attached to the bowel too forcefully will make the bowel wall thinner and increase the risk of perforation.

The largest tumor size in our series measured by CT was 68 * 57 mm, but only a 9-cm serosal defect length was measured, and no mucosal defect was observed after tumorectomy. Two patients with mucosal defects had medium-sized tumors. The size of the tumor fixed in the pelvis at first sight or measured by CT is not the most important factor for considering bowel sparing surgery since the tumor size is not directly related to the depth of bowel infiltration. The depth and width of bowel infiltration are the most important factors, but they cannot be evaluated accurately preoperatively only by CT scan. Several studies in the past have also demonstrated a significant discrepancy between the CT and the surgical findings on bowel involvement (16, 30, 31). In the past decades, a few groups have introduced an exploratory laparoscopy (EXL) before laparotomy (32–35). The advantages of EXL are multiple, including a correct diagnosis based on the histology of tissue biopsy, precise evaluation of disease spread, and better selection of the patients for ultra-radical surgery. The combination of CT and EXL displayed a better diagnostic power on the large bowel involvement than the CT scan alone. Also, it can reliably anticipate the absence of bowel involvement (36). So this preoperative workout should be considered as a method to better discriminate which patients might be eligible for rectosigmoid sparing or resection in our further study. What is more, if a large tumor is packed in the pelvis, even without severe bowel infiltration, there will be no space to perform a tumorectomy since this procedure requires space to clearly expose the bottom of the false capsule and adjust the cutting direction by retracting the tumor in different directions. Presacral space dissection may help to expose the tumor bed under clear visualization.

If mucosal defects occur, two-layer repair should be performed, and sutures should be placed perpendicular to the long axis of the bowel for mucosal repair. Seromuscular defects occurred over a much larger area. There are limited reports on the method of seromuscular repair since bowel resection has been performed in most cases when a muscular invasion was noted. The length of the seromuscular defects in our series was between 3 and 20 cm, and the edges of the defects were irregular. Since the defects were long in some cases and perpendicular repair to the long axis would cause the bowel to fold together, we repaired the defects in an oblique manner to avoid lumen stricturing and bowel folding, which may be safe and effective. Soo et al. (37) reported another safe method of seromuscular repair that formed the rectosigmoid colon into a U-shaped loop, but the lengths of the defects in their reports were 18 cm or less. In particular, the same method of tumorectomy and bowel repair has also been used in upper abdominal surgery in selected cases in our center when seromuscular involvement of the colon caused by omental cake occurred.

In summary, the rectosigmoid sparing en bloc pelvic resection technique described herein may be safe and effective for complete resection in select cases of fixed ovarian tumors infiltrating the colon wall. However, in order to observe the site of recurrence after this procedure, a longer follow-up period is needed, and also, further larger prospective studies are needed to better assess the safety, feasibility, and, most importantly, efficacy in terms of oncological outcomes of this conservative surgical approach.

## Data availability statement

The original contributions presented in the study are included in the article/Supplementary Material. Further inquiries can be directed to the corresponding author.

## Ethics statement

The studies involving human participants were reviewed and approved by Ethics Committee of PUMCH. The patients/participants provided their written informed consent to participate in this study. Written informed consent was obtained from the individual(s) for the publication of any potentially identifiable images or data included in this article.

## Author contributions

Study conceptualization: LP and YJ; Study design: YS and YJ; Data acquisition: YS, YJ, YG, WW, and YL; Quality control of data and algorithms: YJ and YS; Data analysis and interpretation: YS, YL, and YJ; Statistical analysis: YS; Manuscript preparation: YS and YJ; Manuscript editing: all authors; Manuscript review: LP. All authors contributed to the article and approved the submitted version.

## Funding

This project was supported by the Non-profit Central Research Institute Fund of Chinese Academy of Medical Sciences (2021- PT320-003). Furthermore, this project was also supported by CAMS Innovation Fund for Medical Sciences (CIFMS-2017- I2M-1-002).

## Conflict of interest

The authors declare that the research was conducted in the absence of any commercial or financial relationships that could be construed as a potential conflict of interest.

## Publisher’s note

All claims expressed in this article are solely those of the authors and do not necessarily represent those of their affiliated organizations, or those of the publisher, the editors and the reviewers. Any product that may be evaluated in this article, or claim that may be made by its manufacturer, is not guaranteed or endorsed by the publisher.
